# EUCAST Susceptibility Testing of Isavuconazole: MIC Data for Contemporary Clinical Mold and Yeast Isolates

**DOI:** 10.1128/AAC.00073-19

**Published:** 2019-05-23

**Authors:** Karin Meinike Jørgensen, Karen Marie Thyssen Astvad, Rasmus Krøger Hare, Maiken Cavling Arendrup

**Affiliations:** a Unit of Mycology, Statens Serum Institut, Copenhagen, Denmark; b Department of Clinical Microbiology, Rigshospitalet, Copenhagen, Denmark; c Department of Clinical Medicine, University of Copenhagen, Copenhagen, Denmark

**Keywords:** *Aspergillus*, *Candida*, EUCAST, Mucorales, antifungal susceptibility testing, azoles

## Abstract

Isavuconazole is the newest medical azole. We investigated EUCAST MICs for isavuconazole and seven comparators against 1,498 contemporary isolates (2016 to 2017). EUCAST susceptibility testing was performed. Isavuconazole MICs >2 dilution steps above the modal MIC were regarded as non-wild type for species without EUCAST epidemiological cutoff values (ECOFFs). *CYP51A* sequencing was performed when relevant. Pearson correlation analysis was adopted for comparing activity. *Aspergillus* accounted for 90% of mold and *Candida* accounted for 97% of yeast isolates. Thirty (9.3%) Aspergillus fumigatus isolates were classified as resistant, and 10 (3.1%) were classified as non-wild type. Thirteen (4%) were cross-resistant to other mold-active azoles. Target gene alterations were found in 10 (76.9%) isolates, including 4 (30.8%) of environmental origin (TR_34_/L98H [*n* = 3] and Trip_34_^3^/L98H [*n* = 1]). Six Aspergillus terreus isolates were resistant, including two (17%) with MICs of >2 mg/liter and M217I alterations. Modal MICs/MIC_50_s (milligrams per liter) against *Candida* spp. were ≤0.004/≤0.004 for C. albicans and C. dubliniensis, 0.008/0.008 for C. tropicalis, 0.016/0.016 for C. parapsilosis, 0.06/0.06 for C. glabrata, and 0.125/0.125 for C. krusei. A non-wild-type phenotype was observed for 6.6% of isolates (C. glabrata [11.8%] and C. tropicalis [12.3%], specifically). All of these isolates were nonsusceptible/non-wild type to fluconazole (96.1%) or voriconazole (86.2%). Low MICs were found for several other species, except Scedosporium apiospermum and *Fusarium*. The best correlation was found between isavuconazole and voriconazole overall but for A. terreus and Mucorales to itraconazole and posaconazole, respectively. Isavuconazole displayed broad *in vitro* activity. Acquired resistance was infrequent except in A. terreus, C. glabrata, and C. tropicalis and, when present, was associated with cross-resistance to other azoles. Revising the EUCAST breakpoints for A. fumigatus (defining an MIC of 2 mg/liter as intermediate [“I”]) would minimize major errors.

## Abstract (precis)

Isavuconazole is the newest medical azole. We investigated EUCAST MICs for isavuconazole and seven comparators against 1,498 contemporary isolates (2016 to 2017).

## INTRODUCTION

Isavuconazole is the newest medical azole with activity against a broad range of yeast and molds. It was licensed in Europe and the United States in 2015 by the EMA and FDA for the treatment of adults with invasive aspergillosis and also for mucormycosis although by the EMA only in patients for whom amphotericin B is inappropriate. In the same year, EUCAST clinical breakpoints were established for three *Aspergillus* species (A. fumigatus, A. nidulans, and A. terreus), and epidemiological cutoff values (ECOFFs) for these as well as for A. flavus and A. niger were determined (1). Isavuconazole given daily or weekly has also been found efficacious and noninferior to fluconazole for uncomplicated esophageal candidiasis in a randomized, double-blind, multicenter phase 2 trial, where Candida albicans was the most common cause of infection, accounting for 94.8% (2). In line with this *in vivo* efficacy, isavuconazole has potent *in vitro* activity against particularly C. albicans, C. parapsilosis, and C. tropicalis, with an MIC of ≤0.03 mg/liter for 91.5% to 96.0% of such isolates (3). MICs against C. glabrata and C. krusei are higher, with modal MICs of between 0.03 mg/liter and 0.5 mg/liter in most data sets (3–6). Unfortunately, EUCAST ECOFFs and breakpoints have not been established for *Candida* species due to significant interlaboratory variability (3).

Previous studies have shown a correlation between the susceptibility to the azoles and, particularly, the susceptibility to voriconazole and isavuconazole for A. fumigatus (7, 8). Similarly, studies have shown that isavuconazole susceptibility was lower for *Candida* isolates with resistance or non-WT (wild-type) susceptibility to fluconazole and voriconazole (9).

Denmark is a high-incidence country for candidemia, with a high use of antifungal compounds, including azoles, in a Nordic perspective (10). Hence, it is of utmost importance to monitor the susceptibility profiles of clinically relevant fungal isolates. In this study, we investigated and compared the *in vitro* activities of isavuconazole and seven comparators against a large contemporary clinical collection of mold and yeast isolates received at the Danish mycology reference center during the years 2016 to 2017. MICs were interpreted by applying recently established EUCAST clinical breakpoints and ECOFFs.

## RESULTS

During 2016 and 2017, isavuconazole susceptibility was determined for 429 mold isolates and 1,069 yeast isolates from 1,325 patients. The MICs for isavuconazole and comparators (voriconazole, itraconazole, posaconazole, and amphotericin B for molds and voriconazole, fluconazole, amphotericin B, anidulafungin, and micafungin for yeasts) were evaluated separately for 2016 and 2017, but as no difference was seen for the 2 years (modal MICs and MIC_50_s within ±1 dilution step [data not shown]), data were pooled and are presented together.

### Molds.

*Aspergillus* accounted for 90.2% (*n* = 387) of the 429 mold isolates (Table 1). Adopting the EUCAST ECOFFs available for the five most prevalent *Aspergillus* complex isolates, 24 (6.4%) were non-wild type, and among the three species for which clinical breakpoints have been established, 36/335 (10.7%) isolates were classified as resistant (R). In detail, 30/322 (9.3%) A. fumigatus isolates (from 28 patients) were classified as resistant, and 10/322 (3.1%) from 9 patients were classified as non-wild type. Three (15%) A. fumigatus isolates with isavuconazole MICs of 2 mg/liter (classified as resistant but within the wild-type range) were cross-resistant to itraconazole (MIC ≥16 mg/liter). Two of these isolates harbored Cyp51A alterations and were also cross-resistant to either posaconazole and voriconazole (G54A; MICs of ≥4 mg/liter and 2 mg/liter, respectively) or posaconazole only (M220K; MIC of ≥4 mg/liter). All 10 A. fumigatus isolates with MICs of >2 mg/liter (and, thus, both resistant and non-wild type) were nonsusceptible to itraconazole, posaconazole, and voriconazole. Eight of these isolates (80%) harbored Cyp51A alterations (TR_34_/L98H [*n* = 3], Trip_34_^3^/L98H [*n* = 1], TR_120_/F46Y/M172V/E427K [*n* = 1], G432S [*n* = 1], and G448S [*n* = 2]). Thus, overall, 13/322 (4%) of the A. fumigatus isolates were classified as isavuconazole resistant and cross-resistant to other mold-active azoles, and 10/13 (76.9%) harbored target gene alterations, including 4/13 (30.8%) whose alterations were due to an environmental resistance mechanism.

**TABLE 1:**
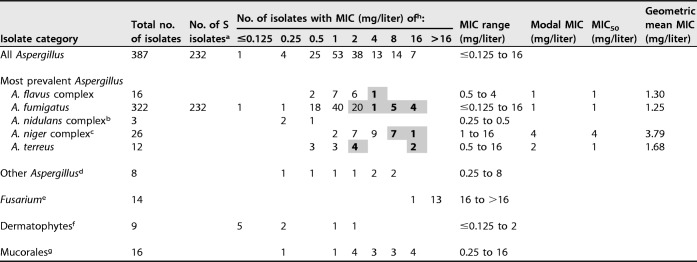
MICs and geometric mean MICs of isavuconazole against the 429 mold isolates

^a^Number of isolates sensitive to itraconazole, posaconazole, and voriconazole determined by azole agar screening.

^b^One isolate each of the A. nidulans complex, A. quadrilineatus, and A. spinulosporus.

^c^Twenty-two isolates of the A. niger complex, three isolates of A. tubingensis, and one of A. luchuensis.

^d^Three A. calidoustus isolates and one isolate each of A. fischeri, A. giganteus, A. persii, A. similis, and A. turcosus.

^e^Two F. dimerum, two F. proliferatum, seven F. solani *sensu stricto*, and three F. solani complex isolates.

^f^Two M. canis, four T. rubrum, one T. interdigitale, and two T. mentagrophytes complex isolates.

^g^Two M. circinelloides isolates, three R. pusillus isolates, four R. microsporus isolates, and one isolate each of C. muscae, L. corymbifera, L. ramosa, *Lichtheimia* species, R. oryzae, Syncephalastrum racemosum, and Mucorales species.

^h^Resistant *Aspergillus* isolates are shaded, and non-wild-type isolates are shown in boldface type.

Six A. terreus (50%) isolates from five patients were classified as isavuconazole resistant and non-wild type, including 4/4 isolates with MICs of 2 mg/liter, the wild-type *CYP51A* target gene, and susceptibility to the other three azoles. The remaining two isolates (17%), with an isavuconazole MIC of >2 mg/liter, harbored an M217I alteration (corresponding to the M220I alteration in A. fumigatus).

Finally, among other molds, low MICs (≤0.25 mg/liter) were observed against Microsporum canis, Trichophyton rubrum, Trichophyton interdigitale, Circinella muscae, and Saprochaete capitata, and high MICs (≥16 mg/liter) were observed against Scedosporium apiospermum, *Fusarium* spp. (including F. dimerum, F. proliferatum, and F. solani complex isolates), and 4/16 Mucorales isolates (Mucor circinelloides [*n* = 3] and Rhizopus oryzae). However, numbers were low.

The *in vitro* activity of isavuconazole against A. flavus, A. fumigatus, A. nidulans, and A. terreus isolates on a milligram-per-liter basis was comparable to those of voriconazole and amphotericin B and slightly lower than those of itraconazole and posaconazole (Table 2). For A. niger isolates, the isavuconazole modal MIC and MIC_50_ were two 2-fold dilutions higher than those for voriconazole and itraconazole and four 2-fold dilutions higher than those for posaconazole and amphotericin B. As isavuconazole and the comparators are all regarded as valid options for A. fumigatus infections, the proportions of isolates with MICs above the ECOFF against A. fumigatus were compared for each compound as an indicator of relative coverage (Table 2). The proportions of A. flavus and A. terreus isolates with MICs above the A. fumigatus ECOFF were lower for isavuconazole than for amphotericin B and voriconazole, whereas in contrast, the proportion of A. niger isolates less susceptible than A. fumigatus was highest for isavuconazole (Table 2).

**TABLE 2:** Modal MICs/MIC_50_s and MIC ranges for isavuconazole and comparators for the 429 mold isolates^*h*^

Isolate category	Total no. of isolates	Isavuconazole		Voriconazole		Itraconazole		Posaconazole		Amphotericin B	
		Modal MIC/MIC_50_ (range) or MIC range (mg/liter)	% of isolates with MIC > 2 mg/liter	Modal MIC/MIC_50_ (range) or MIC range (mg/liter)	% of isolates with MIC > 1 mg/liter	Modal MIC/MIC_50_ (range) or MIC range (mg/liter)	% of isolates with MIC > 1 mg/liter	Modal MIC/MIC_50_(range) or MIC range (mg/liter)	% of isolates with MIC > 0.25 mg/liter	Modal MIC/MIC_50_ (range) or MIC range (mg/liter)	% of isolates with MIC > 1 mg/liter
All *Aspergillus*	387	1/1 (≤0.125 to 16)	8.9	1/1 (0.25 to 16)	8.5	0.25/0.5 (≤0.125 to >16)	9.8	0.125/0.125 (≤0.03 to >4)	8.80	0.5/0.5 (0.03 to >4)	3.1

Most prevalent *Aspergillus*											
A. flavus complex	16	1/1 (0.5 to 4)	6.3	1/1 (0.5 to 2)	12.5	0.25/0.25 (≤0.125 to 0.5)	0	0.125/0.125 (0.06 to 0.25)	0	1/1 (0.5 to 4)	18.8
A. fumigatus	322^*a*^	1/1 (≤0.125 to 16)	3.1	0.5/0.5 (0.25 to 16)	4.0	0.25/0.25 (≤0.125 to >16)	6.8	0.125/0.125 (≤0.03 to >4)	6.2	0.5/0.5 (0.125 to 1)	0
A. nidulans complex^*b*^	3	0.25 to 0.5	0/3	0.25 to 0.5	0/3	≤0.125 to 0.5	0/3	0.06 to 0.5	1/3	0.25 to 1	0/3
A. niger complex^*c*^	26	4/4 (1 to 16)	65.4	1/1 (0.5 to 4)	42.3	1/1 (0.25 to >16)	38.5	0.25/0.25 (≤0.03 to 0.5)	23.1	0.25/0.25 (0.03 to 0.5)	0
A. terreus	12	2/1 (0.5 to 16)	16.7	0.5/1 (0.5 to 8)	25	≤0.125/0.25 (≤0.125 to >16)	16.7	0.06/0.06 (≤0.03 to 0.5)	16.7	1/1 (1 to 4)	50

Other *Aspergillus*^*d*^	8	0.25 to 8	50	0.25 to 8	50	≤0.125 to >16	50	0.06 to >4	62.5	0.125 to >4	25

*Fusarium*^*e*^	14	(16 to >16)	100	(4 to >16)	100	(4 to >16)	100	>4	100	0.5 to >4	38.5

Dermatophytes^*f*^	9	≤0.125 to 2	0/9	≤0.125 to 1	0/9	≤0.125 to >16	3/9	≤0.03 to 0.5	2/9	0.25 to 1	0/8
											
Mucorales^*g*^	16	0.25 to 16	62.5	8 to >16	100	≤0.125 to >16	60	≤0.03 to >4	68.8	0.03 to 2	6.3

a A total of 232/322 A. fumigatus isolates were determined to be sensitive to itraconazole, posaconazole, and voriconazole by azole agar screening.

b One isolate each of the A. nidulans complex, A. quadrilineatus, and *A. spinulosporus*.

c Twenty-two A. niger complex, three A. tubingensis, and one *A. luchuensis* isolate.

d Three A. calidoustus isolates and one isolate each of *A. fischeri*, A. giganteus, *A. persii*, *A. similis*, and *A. turcosus*.

e Two F. dimerum, two F. proliferatum, seven F. solani *sensu stricto*, and three F. solani complex isolates.

f Two *M. canis*, four T. rubrum, one T. interdigitale, and two T. mentagrophytes complex isolates.

g Two *M. circinelloides* isolates, three *R. pusillus* isolates, four R. microsporus isolates, and one isolate each of *C. muscae*, L. corymbifera, L. ramosa, *Lichtheimia* species, *R. oryzae*, S. racemosum, and Mucorales species.

h The proportions of isolates with MICs above the A. fumigatus EUCAST ECOFF are illustrated.

### Yeast.

*Candida* accounted for 97.2% (*n* = 1039) of the 1,069 yeast isolates (Table 3). Other species were Saccharomyces cerevisiae (12; 1.1%), Cryptococcus neoformans (10; 0.9%), and rare yeast (8; 0.7%). Overall, 91% of MICs fell at ≤0.125 mg/liter, and 95% were ≤0.25 mg/liter.

**TABLE 3:**
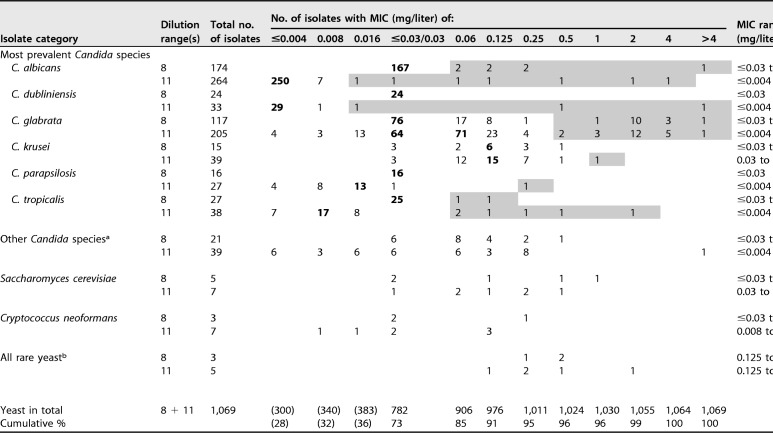
MICs and geometric means of isavuconazole against the 1,069 yeast isolates^*c*^

^a^Fifteen C. lusitaniae isolates; 11 C. guilliermondii isolates; 6 C. orthopsilosis isolates; 5 C. kefyr isolates; 4 isolates of C. fermentati, C. inconspicua, and C. pelliculosa; 2 C. norvegensis isolates; and 1 isolate each of C. auris, C. bovina, C. duobushaemulonii, C. famata, C. metapsilosis, C. nivariensis, C. palmioleophila, C. pararugosa, and C. utilis.

^b^Two Geotrichum candidum and Rhodotorula mucilaginosa isolates and one isolate each of Arxula adeninivorans, Exophiala dermatitidis, Geotrichum silvicola, and Magnusiomyces capitatus.

c Modal MICs are highlighted in boldface type for species with ≥10 isolates, and presumably non-wild-type MIC ranges are shaded for *Candida* isolates with MICs >2 dilution steps above the modal MIC (≥2 dilution steps when the MIC distribution was clearly truncated with the modal MIC at the lowest dilution tested [0.004 mg/liter]). The numbers and percentages in parentheses represent underestimates of the true numbers and proportions due to the truncation of the tested drug concentration ranges.

The vast majority of the MICs against the most susceptible *Candida* species fell at or below the lowest concentrations tested (Table 3), i.e., for C. albicans, 417/438 (95.2%) overall and 250/264 (94.7%) for the extended concentration range, specifically, and for C. dubliniensis, 53/57 (93.0%) and 29/33 (87.9%), respectively. Consequently, modal MIC and MIC_50_ values were ≤0.004 mg/liter. For the other common *Candida* species, the modal MIC/MIC_50_ values (milligrams per liter) were as follows: 0.008/0.008 for C. tropicalis, 0.016/0.016 for C. parapsilosis, 0.06/0.06 for C. glabrata, and 0.125/0.125 for C. krusei. In all cases, the modal MIC and MIC_50_ values set using the 11-dilution range were supported by the 8-dilution range adopted in the first part of the study period, suggesting a robust performance of EUCAST isavuconazole testing during the study period.

For 65/979 (6.6%) isolates from these six most prevalent *Candida* species (isolated from 60 patients), an isavuconazole MIC >2 dilution steps above the modal MIC for a given species (≥2 dilutions if the modal MIC fell at the lowest concentration tested [0.004 mg/liter]) could be documented, suggesting a non-wild-type phenotype (indicated with gray shading in Table 3). In detail, this was the case for 14/438 (3.2%) C. albicans, 3/57 (5.3%) C. dubliniensis, 38/322 (11.8%) C. glabrata, 1/54 (1.9%) C. krusei, 1/43 (2.3%) C. parapsilosis, and 8/65 (12.3%) C. tropicalis isolates. When fluconazole and voriconazole susceptibilities were investigated for these isolates, 96.1% were nonsusceptible/non-wild type to fluconazole, 86.2% were nonsusceptible/non-wild type to voriconazole, and all were nonsusceptible/non-wild type to at least one of the two (data not shown). For C. glabrata, 13/38 (34.2%) of the isavuconazole non-WT isolates presented fluconazole MICs of >16 mg/liter, rendering classification as intermediate (I) or R impossible. One of these isolates was voriconazole susceptible (S), whereas the remaining 12 were classified as voriconazole non-WT.

The rare *Candida* species were characterized by isavuconazole MICs spanning the entire ≤0.004- to >4-mg/liter concentration range, suggesting differential susceptibilities of the involved species. The most resistant species was *C. duobushaemulonii* (*n* = 1), with an MIC of >4 mg/liter, whereas the single C. auris isolate, originating from a Norwegian patient, as well as isolates belonging to *C. bovina*, C. famata, C. kefyr, C. metapsilosis, C. nivariensis, *C. palmioleophila*, *C. pararugosa*, C. pelliculosa, and *C. utilis* had isavuconazole MICs of ≤0.06 mg/liter. Similarly, the MICs for rare yeast were diverse, with Magnusiomyces capitatus being the isolate with the highest isavuconazole MIC of 2 mg/liter.

On a milligram-per-liter basis, the *in vitro* activity of isavuconazole was more similar to that of voriconazole against the yeast isolates than to that of fluconazole and similar to that of the echinocandins, except for C. parapsilosis, *Cryptococcus*, C. glabrata, and C. krusei (Table 4).

**TABLE 4:** Modal MICs/MIC_50_s and proportions of non-wild-type isolates for isavuconazole and comparators for the yeast isolates^*e*^

Isolate category	Dilution range	Total no. of isolates	ISA		FLC		VRC		AMB^*a*^		ANF		MFG	
			Modal MIC/MIC_50_ (range) or range (mg/liter)	% of non-WT isolates	Modal MIC/MIC_50_ (range) or range (mg/liter)	% of non-WT isolates	Modal MIC/MIC_50_ (range) or range (mg/liter)	% of non-WT isolates	Modal MIC/MIC_50_ (range) or range (mg/liter)	% of non-WT isolates	Modal MIC/MIC_50_ (range) or range (mg/liter)	% of non-WT isolates	Modal MIC/MIC_50_ (range) or range (mg/liter)	% of non-WT isolates
C. albicans	8	174	≤0.03/≤0.03 (≤0.03 to >4)	3.2	0.25/0.25 (≤0.125 to >16)	2.7	≤0.03/≤0.03 (≤0.03 to 2)	2.5	ND	0	≤0.008/≤0.008 (≤0.008 to 0.03)	0.3	≤0.008/≤0.008 (≤0.008 to 0.03)	3.2
	11	264	≤0.004/≤0.004 (≤0.004 to 4)		0.25/0.25 (0.03 to >32)		≤0.004/≤0.004 (≤0.004 to 2)		0.25/0.25 (≤0.016 to 0.5)		≤0.004/≤0.004 (≤0.004 to 0.125)		0.008/0.008 (≤0.004 to 0.5)	

C. dubliniensis	8	24	≤0.03/≤0.03 (≤0.03)	5.3	≤0.125/≤0.125 (≤0.125 to 4)	5.3	≤0.03/≤0.03 (≤0.03)	5.3	ND	0	0.016/0.016 (≤0.008 to 0.5)	2.2	0.016/0.016 (≤0.008 to 1)	6.5
	11	33	≤0.004/≤0.004 (≤0.004 to >4)		0.125/0.25 (0.06 to >32)		0.008/0.008 (≤0.004 to >4)		0.03/0.03 (≤0.016 to 0.25)		0.008/0.008 (≤0.004 to 0.03)		0.016/0.016 (≤0.004 to 0.06)	

C. glabrata	8	117	≤0.03/≤0.03 (≤0.03 to >4)	11.8	4/4 (0.5 to >16)	7.8^*d*^	0.06/0.06 (≤0.03 to 4)	7.8	ND	0	0.06/0.06 (0.016 to 1)	1.9	≤0.008/≤0.008 (≤0.008 to 1)	2.7
	11	205	0.06/0.06 (≤0.004 to >4)		4/4 (0.5 to >32)		0.06/0.06 (0.016 to >4)		0.25/0.25 (0.03 to 1)		0.016/0.016 (0.008 to 1)		0.016/0.016 (≤0.004 to 0.5)	

C. krusei	8	15	0.125/0.125 (≤0.03 to 0.5)	1.9	>16/>16 (16 to >16)	0	0.25/0.25 (0.125 to 1)	1.9	ND	0	0.06/0.06 (0.03 to 0.06)	0	0.125/0.125 (0.06 to 0.25)	0
	11	39	0.125/0.125 (0.03 to 1)		32/32 (8 to >32)		0.25/0.25 (0.125 to 2)		0.5/0.5 (0.5 to 1)		0.03/0.03 (0.016 to 0.06)		0.125/0.125 (0.06 to 0.5)	

C. parapsilosis	8	16	≤0.03/≤0.03 (≤0.03)	2.3	1/1 (≤0.125 to 4)	8.7	≤0.03/≤0.03 (≤0.03 to 0.06)	3.3	ND	0	>1/>1 (1 to >1)	0	(1/>1)/>1 (1 to >1)	0
	11	27	0.016/0.016 (≤0.004 to 0.25)		(0.5/1)/1 (0.5 to >32)		0.016/0.016 (0.008 to 2)		0.5/0.5 (0.125 to 1)		0.5/1 (0.5 to 2)		2/2 (0.5 to 4)	

C. tropicalis	8	27	≤0.03/≤0.03 (≤0.03 to 0.125)	12.3	0.5/0.5 (≤0.125 to 16)	10.8	≤0.03/≤0.03 (≤0.03 to 0.25)	12.3	ND	0	0.03/0.03 (≤0.008 to 0.5)	1.0	0.016/0.016 (≤0.008 to 0.5)	1.0
	11	38	0.008/0.008 (≤0.004 to 2)		0.5/0.5 (0.125 to >32)		0.016/0.03 (0.008 to >4)		0.25/0.25 (0.125 to 1)		0.016/0.016 (≤0.004 to 0.03)		0.03/0.03 (≤0.004 to 0.06)	

Other *Candida* species^*b*^	8	21	≤0.03 to 0.5		≤0.125 to >16		≤0.03 to 2				0.016 to >1		0.03 to 0.5	
	11	39	≤0.004 to >4		0.06 to >32		≤0.004 to >4		0.06 to >4		≤0.004 to 2		0.016 to 0.5	

Saccharomyces cerevisiae	8	5	≤0.03 to 1		2 to >16		0.06 to 0.5				0.06 to 0.25		0.125 to 0.25	
	11	7	0.03 to 0.5		2 to 16		0.06 to 0.5		0.25/0.25 (0.06 to 1)		0.016 to 0.125		0.06 to 0.125	

Cryptococcus neoformans	8	3	≤0.03 to 0.25		1 to 16		≤0.03 to 0.25				>1		>1	
	11	7	0.008 to 0.125		2 to 16		0.016 to 0.125		0.25/0.25 (0.03 to 0.5)		>4		>4	

All rare yeast^*c*^	8	3	0.125 to 0.5		8 to >16		0.06 to 2				>1		>1	
	11	5	0.125 to 2		16 to >32		0.25 to >4		0.06 to 1		0.125 to >4		0.03 to >4	

a Amphotericin B was tested only in a wide concentration range.

b Fifteen C. lusitaniae isolates; 11 C. guilliermondii isolates; 6 C. orthopsilosis isolates; 5 C. kefyr isolates; 4 isolates of *C. fermentati*, C. inconspicua, and C. pelliculosa; 2 C. norvegensis isolates; and one isolate each of C. auris, *C. bovina*, *C. duobushaemulonii*, C. famata, C. metapsilosis, C. nivariensis, *C. palmioleophila*, *C. pararugosa*, and *C. utilis*.

c Two Geotrichum candidum and Rhodotorula mucilaginosa isolates and one isolate each of *Arxula adeninivorans*, *Exophiala dermatitidis*, *Geotrichum silvicola*, and Magnusiomyces capitatus.

d Potentially an underestimate of the true non-wild-type proportion, as some of the C. glabrata isolates were not tested at fluconazole concentrations above 16 mg/liter.

e ISA, isavuconazole; FLC, fluconazole; VRC, voriconazole; AMB, amphotericin B; ANF, anidulafungin; MFG, micafungin; ND, not done.

### Correlation between susceptibilities to isavuconazole and comparators.

Finally, the correlation between isavuconazole and comparator MICs was investigated (Table 5). A significant correlation was observed between isavuconazole and voriconazole MICs for all mold and *Candida* species, although it was weak (*R*^2^ of <0.5 for Mucorales, C. krusei, and C. parapsilosis). Moreover, the correlation between isavuconazole and voriconazole was stronger than that between isavuconazole and any other comparator for all species except A. terreus and Mucorales species (the best correlation was observed for isavuconazole and posaconazole). A good and highly significant correlation was also observed between isavuconazole and fluconazole for C. albicans, C. glabrata, and C. tropicalis but not for C. parapsilosis.

**TABLE 5:** Correlation between isavuconazole MICs and those of voriconazole, itraconazole, posaconazole, and amphotericin B^*a*^

Species	Voriconazole		Itraconazole		Posaconazole		Fluconazole		Amphotericin B		No. of isolates^*b*^
	*R*^2^	*P*	*R*^2^	*P*	*R*^2^	*P*	*R*^2^	*P*	*R*^2^	*P*	
A. flavus	**0.598**	0.0004	0.090	0.2581	0.196	0.086	ND	ND	0.229	0.0608	16
A. fumigatus	**0.766**	<0.0001	0.289	<0.0001	0.071	0.0115	ND	ND	0.003	0.6276	89
A. niger	**0.573**	<0.0001	0.537	0.0002	0.225	0.0177	ND	ND	0.0414	0.3405	21–24
A. terreus	0.740	0.0003	0.763	0.0004	0.807	0.0002	ND	ND	0.585	0.0038	10–12

Mucorales	0.381	0.0108	0.072	0.3336	0.691	<0.0001	ND	ND	0.101	0.2291	15–16

C. albicans	**0.896**	<0.0001	ND	ND	ND	ND	0.786	<0.0001	0.0215	0.4564	9–28
C. dubliniensis	**0.938**	0.0015	ND	ND	ND	ND	0.857	0.008	0.675	0.0234	6–7
C. glabrata	**0.862**	<0.0001	ND	ND	ND	ND	0.817	<0.0001	0.0092	0.1361	216–244
C. krusei	**0.417**	<0.0001	ND	ND	ND	ND	0.207	0.0008	0.0146	0.3983	51
C. parapsilosis	**0.448**	0.0002	ND	ND	ND	ND	0.00871	0.6573	0.0934	0.1289	25–26
C. tropicalis	**0.786**	<0.0001	ND	ND	ND	ND	0.668	<0.0001	0.0679	0.1306	33–35

Other C*andida*	**0.514**	<0.0001	ND	ND	ND	ND	0.439	<0.0001	0.000464	0.8845	42–48

a The strongest correlation (highest *R*^2^ coefficient) for each species across the four comparators is highlighted in boldface type, and significant *P* values are underlined. ND, not done.

b Number of paired values for comparison (isolates with MICs below the lowest concentration were excluded from analyses, whereas isolates with MICs above the concentration range were elevated to the nearest higher 2-fold dilution).

## DISCUSSION

This study confirmed previous findings of potent *in vitro* activity of isavuconazole against most human-pathogenic fungi (3–5, 9, 11). The rate of acquired resistance in *Aspergillus* and *Candida* spp. was overall low and stable (4). Exceptions were resistance rates of around 10% in A. fumigatus, A. terreus, C. glabrata, and C. tropicalis. However, resistance rates may be overestimated, as fungal isolates referred to a reference laboratory may not be representative of the general population. For the two *Aspergillus* species, however, the resistance was in part due to a stringent susceptibility breakpoint bisecting the wild-type populations of A. fumigatus and A. terreus, leading to a misclassification of some susceptible isolates as resistant. The ECOFF for isavuconazole against A. fumigatus is 2 mg/liter, but the clinical breakpoint established was one step lower because isolates with an MIC of 2 mg/liter may represent isolates with wild-type as well as mutant target gene sequences (1, 3). Thus, in a multicenter study, MICs straddled the ECOFF, with MICs of >2 mg/liter found in 25% of isolates harboring the M220I and M220V mutations and in 72.5% of the MIC readings of isolates withTR_34_/L98H alterations, suggesting a slight and more prominent reduction of susceptibility. The majority of the A. fumigatus isolates classified as wild type but resistant due to an MIC of 2 mg/liter in the present study had no cross-resistance to other azoles and no target gene alterations. Moreover, one isolate was voriconazole susceptible and harbored the M220K alteration, which previously has been shown not to affect isavuconazole susceptibility (3). Finally, one strain harbored a G54A alteration and was voriconazole intermediate. Whereas isavuconazole susceptibility in strains harboring G54E, G54R, G54V, or G54W alterations appears unaffected, to our knowledge, it is not known if G54A may affect isavuconazole and voriconazole susceptibility (3). Hence, our data suggested that the majority of isolates with an MIC of 2 mg/liter will be true wild-type isolates, and the remaining minority will be characterized by slightly reduced isavuconazole susceptibility. Buil et al. found that the probability of target attainment for isolates with isavuconazole MICs of 2 mg/liter with the isavuconazole standard dose was ∼75% (64% to 92%) when the 90% exposure index (EI_90_) was used as the endpoint and that a trough level of ≥1.60 mg/liter (1.42 to 1.80 mg/liter) was the target (8). Another recent study reported that approximately 10% of “real-life” clinical samples contained less than 1 mg/liter and another approximately 20% contained between 1 and 2 mg/liter of isavuconazole (12). Taken together, these observations support introducing an intermediate category for A. fumigatus and A. terreus isolates with an MIC of 2 mg/liter in a setting where therapeutic drug monitoring is available to confirm sufficient exposure.

When the correlations between isavuconazole MICs and those for the comparators were analyzed, the strongest correlation overall was found for isavuconazole and voriconazole. Thus, a significant strong to moderate correlation was found for the four most common *Aspergillus* species as well as for the six most common *Candida* species except C. krusei and C. parapsilosis, for which the correlation was significant but weak (*R*^2^ of 0.417 to 0.448), potentially due to the lack of isolates with acquired resistance for these two species. Thus, our results extend previous findings of a correlation between the azoles and, particularly, voriconazole and isavuconazole for A. fumigatus (7, 8) and the findings that isavuconazole susceptibility was lower for *Candida* isolates with resistance or non-wild-type susceptibility to fluconazole and voriconazole (9). No correlation was found for isavuconazole compared to itraconazole or posaconazole for A. flavus or A. fumigatus. For A. fumigatus, this may not be surprising, as it is well acknowledged that some target gene mutations specifically affect itraconazole and posaconazole (3). For A. flavus, we assume that the absence of isolates with differential susceptibility explains the lack of correlation because MIC variation in such cases can be explained solely by test variation. Therefore, taken together, our data support that voriconazole susceptibility is a strong marker of isavuconazole susceptibility in most clinically relevant *Candida* and *Aspergillus* isolates. Of note, this suggests that the azole agar screening method (EUCAST E.Def 10.1) can be adopted for identification of A. fumigatus isolates suitable as targets for isavuconazole therapy despite the fact that an isavuconazole agar is not included in the plate design (13).

Isavuconazole is licensed as a second-line option for the treatment of Mucorales infections in adults after the VITAL study showed equal clinical efficacy compared to that for matched historical controls treated with amphotericin B (14). In that study, species-specific outcome evaluation was not performed, probably in part because one-third of the cases lacked species identification. We found consistently high MICs of ≥16 mg/liter for *M. circinelloides*, confirming previous findings by CLSI and EUCAST testing (15, 16). Hence, species identification is highly recommended, as clinical efficacy remains to be confirmed for this species.

Isavuconazole is not licensed for the treatment of invasive candidiasis after a recent phase 3, randomized, double-blind, multinational clinical trial failed to demonstrate noninferiority at the end of intravenous (i.v.) therapy compared to caspofungin (17). Thus, the trial supported the results of other clinical studies showing superiority of echinocandins over azoles and amphotericin B and, thus, the recommendation of echinocandins as first-line agents for candidemia and invasive candidiasis (18–21). Nevertheless, the secondary endpoints (overall response to therapy 2 weeks after the end of therapy and all-cause mortality on days 14 and 56) were similar between arms, as were safety and median time to clearance from the bloodstream. On this background and taking the potent *in vitro* activity and attractive safety profile compared to fluconazole and voriconazole into account, isavuconazole might serve as a valid second-line option in settings where echinocandin resistance is likely or documented, mold coverage is indicated, or oral therapy is preferred.

In summary, isavuconazole displayed broad *in vitro* activity against most human-pathogenic species, including dermatophytes and several uncommon species. Of note, however, we confirmed low isavuconazole *in vitro* activity against *M. circinelloides* and therefore advocate for performing species identification, also for Mucorales, whenever possible. Acquired isavuconazole resistance was infrequent, except in A. terreus, C. glabrata, and C. tropicalis, and, when present, was associated with cross-resistance to other azoles. Continued surveillance remains important.

## MATERIALS AND METHODS

### Isolates.

In total, 1,069 yeast and 429 mold isolates from 1,325 patients were included (1 isolate each from 1,186 patients and 2 to 7 isolates from 139 patients). The isolates were prospectively obtained from clinical samples or pure cultures received at the mycology reference laboratory at Statens Serum Institut for identification and susceptibility testing during 2016 and 2017. No ethical restraints apply to studies of routinely obtained anonymized laboratory data. Same-species isolates from the same patient were excluded from the study if sampled ≤21 days apart and identical MICs (within ±1 dilution step) were seen. The isolates derived from the entire country and the following clinical specimens: blood as part of the national surveillance program (915 specimens), airways/lung/pleura/sinus (404), other normally sterile sites (47), urine (20), skin/scalp/nail (24), cervix/vagina/urethra (13), other superficial sites (39), and other/unspecified (36). Yeast identification was done using macro- and micromorphology, supplemented by thermotolerance (incubation at 37°C and 43°C), matrix-assisted laser desorption ionization–time of flight mass spectrometry (Bruker, Bremen, Germany) for *Candida* (22), and, when needed, internal transcribed spacer (ITS) sequencing (23). Similarly, mold identification was done by classical techniques, including thermotolerance (incubation at 50°C) for discriminating A. fumigatus sensu stricto from cryptic species, which underwent β-tubulin sequencing (24). The use of the term “complex” is acknowledged for *Aspergillus* species other than A. fumigatus, in the absence of detailed molecular identification, although for simplicity, it is not used throughout this work. ITS and TEF (transcription elongation factor) sequencing were adopted for other molds and *Fusarium* species, specifically (23, 25).

### Susceptibility testing.

EUCAST susceptibility testing was performed according to E.Def 7.3.1 for yeast. Isavuconazole and amphotericin B MICs were determined for all 1,069 yeast isolates, voriconazole and fluconazole MICs were determined for 1,068/1,069 (99.9%) isolates, micafungin MICs were determined for 1,066/1,069 (99.7%) isolates, and anidulafungin MICs were determined for 1,064/1,069 (99.5%) of the yeast isolates. For the molds, A. fumigatus isolates were screened for azole resistance according to EUCAST E.Def 10.1 using a four-well plate containing RPMI 1640–2% glucose agar supplemented with itraconazole (4 mg/liter), voriconazole (1 mg/liter), posaconazole (0.5 mg/liter), and no antifungal (positive control) (Balis Laboratorium VOF, Boven-Leeuwen, the Netherlands). In brief, 25 μl of a conidial suspension (filtered through an 11-nm filter) at a 0.5 McFarland standard was added to each well, and the plate was incubated for 48 h at 37°C before reading. Screening of agar-positive A. fumigatus isolates and all other molds was performed according to EUCAST E.Def 9.3.1, with standard filtration (11-nm filter) of the inoculum. Isavuconazole and voriconazole susceptibilities were determined for all 429 mold isolates, posaconazole susceptibility was determined for 428/429 (99.8%) isolates, and itraconazole and amphotericin B susceptibilities were determined for 427/429 (99.5%) of the mold isolates. Stock solutions of the following antimycotics were prepared at 5,000 mg/liter in dimethyl sulfoxide (Sigma-Aldrich, Brøndby, Denmark): isavuconazole (Basilea Pharmaceutica Ltd., Basel, Switzerland), voriconazole (Pfizer, Ballerup, Denmark), itraconazole (Sigma-Aldrich), posaconazole (MSD, Ballerup, Denmark), fluconazole (Sigma), amphotericin B (Sigma), anidulafungin (Pfizer, Ballerup, Denmark), and micafungin (Astellas, Tokyo, Japan). Cell culture-treated microtiter polystyrene plates (Nunc microwell 96-well microplates, catalog no. 167008; Thermo Fisher Scientific) were used throughout. Candida krusei ATCC 6258 and Candida parapsilosis ATCC 22019 were used as controls for the yeasts, and Aspergillus flavus ATCC 204304 and Aspergillus fumigatus ATCC 204305 were used as controls for the molds. For the yeast isolates, during the study period, the concentration range was extended from 8 to 11 dilutions. *CYP51A* sequencing was performed for non-wild-type/resistant A. fumigatus and A. terreus isolates.

### Data analysis.

Modal MICs, MIC_50_s, geometric mean MICs (GM-MICs), and MIC ranges were determined for individual species (*n* ≥ 10). EUCAST ECOFFs/breakpoints were adopted for wild-type/susceptibility classification. For species without EUCAST ECOFFs, MICs >2 dilution steps above the modal MIC were regarded as non-wild type. However, for species where the modal MIC was equal to or lower than the lowest concentration tested, MICs ≥2 dilution steps above the modal MIC were regarded as non-wild type. Pearson correlation analyses with a two-tailed *P* value were performed for comparisons of antifungal *in vitro* activities (after log_2_ transformation) using GraphPad Prism 7.04. Correlation coefficients (squared) (*R*^2^) of ≥0.5 with a *P* value <0.05 were interpreted as a significant indicator of good to strong correlation, whereas *R*^2^ values of <0.5 indicated weak correlation.
